# *Paraburkholderia phytofirmans* PsJN colonization of rice endosphere triggers an atypical transcriptomic response compared to rice native *Burkholderia s.l.* endophytes

**DOI:** 10.1038/s41598-023-37314-7

**Published:** 2023-07-03

**Authors:** Eoghan King, Adrian Wallner, Ludivine Guigard, Isabelle Rimbault, Hugues Parrinello, Agnieszka Klonowska, Lionel Moulin, Pierre Czernic

**Affiliations:** 1 Plant Health Institute of Montpellier, IRD, CIRAD, University of Montpellier, l’Institut Agro, Montpellier, France; 2 Montpellier GenomiX (MGX), c/o Institut de Génomique Fonctionnelle, Montpellier, France; 3 Present Address: Centro de Biotecnología y Genómica de Plantas (CBGP), Universidad Politécnica de Madrid (UPM)–Instituto Nacional de Investigación y Tecnología Agraria y Alimentación (INIA/CSIC), Campus de Montegancedo, Pozuelo de Alarcón, Madrid Spain; 4 Present Address: SFR Condorcet – FR CNRS 3417, University of Reims Champagne-Ardenne, Induced Resistance and Plant Bioprotection (RIBP) – EA 4707, Cedex 2, BP1039, 51687 Reims, France

**Keywords:** Microbe, Plant symbiosis, Plant signalling

## Abstract

The plant microbiome has recently emerged as a reservoir for the development of sustainable alternatives to chemical fertilizers and pesticides. However, the response of plants to beneficial microbes emerges as a critical issue to understand the molecular basis of plant-microbiota interactions. In this study, we combined root colonization, phenotypic and transcriptomic analyses to unravel the commonalities and specificities of the response of rice to closely related *Burkholderia s.l.* endophytes. In general, these results indicate that a rice-non-native *Burkholderia s.l.* strain, *Paraburkholderia phytofirmans* PsJN, is able to colonize the root endosphere while eliciting a markedly different response compared to rice-native *Burkholderia s.l.* strains. This demonstrates the variability of plant response to microbes from different hosts of origin. The most striking finding of the investigation was that a much more conserved response to the three endophytes used in this study is elicited in leaves compared to roots. In addition, transcriptional regulation of genes related to secondary metabolism, immunity, and phytohormones appear to be markers of strain-specific responses. Future studies need to investigate whether these findings can be extrapolated to other plant models and beneficial microbes to further advance the potential of microbiome-based solutions for crop production.

## Introduction

The potential of plant-associated microbes is nowadays appearing as a promising alternative solution to reduce the negative impacts caused using chemical pesticides and fertilizers in agriculture. Indeed, the application of beneficial microbes can enhance plant production by improving nutrient uptake or alleviating the impact of environmental stresses through various mechanisms^1^. Of particular interest is the use of endophytes, which are microbes able to colonize the inner tissues of plants and provide beneficial effects to the plants throughout their life cycle. Among the diversity of microbial endophytes, one particular bacterial strain, *Paraburkholderia phytofirmans* PsJN, hereafter termed PsJN, has the ability to colonize the endosphere of numerous plant species such as *Arabidopsis,* potato, tomato, grapevine, maize and barley^2^. PsJN also exhibit multiple beneficial effects to plants, in particular by increasing the tolerance of inoculated plants to biotic and abiotic stresses^3^.

In order to identify the molecular mechanisms implicated in the beneficial effects of PsJN, several studies analyzed the transcriptional regulations of PsJN-treated *Arabidopsis* plants. For direct beneficial effects, PsJN-triggered root growth promotion by involving hormone signaling, particularly auxin and ethylene^4^. Also, systemic transcriptional regulations lead to life cycle shortening through the early up-regulation of flowering control genes^5^. Transcriptional analyses were also carried out in the context of PsJN-induced resistance to pathogens^6,7^, salt^8^ and cold tolerance^9^. However, the interaction between the model endophyte PsJN and rice (*Oryza sativa*) has never been studied.

Furthermore, it appears interesting to compare the plant response to the colonization by broad-spectrum versus rice-natural endophytes to unravel plant–microbe specific interactions. Similar comparative analyses of hosts transcriptomic response have been done to compare the response to symbiont and pathogens^10–12^ and the response to different beneficial strains of *Azospirillum* isolated from different cultivars of rice^13^. Therefore, we analyzed the response of rice to the inoculation with PsJN. Then, we performed a comparative analysis of rice transcriptomic response to PsJN with the response to two rice-isolated *Burkholderia s.l.* endophytic strains that we described in a previous study^14^: the rice-endophytic models *Paraburkholderia kururiensis* M130^15^, hereafter termed Pk, and *Burkholderia vietnamiensis* TVV75, hereafter termed Bv. Our objective was to unravel common and specific signatures of rice transcriptomic response to these three strains to characterize the specificities of the physiological response of rice plants to a diversity of closely related strains.

To study the response of rice to PsJN, we first analyzed the colonization of *Oryza sativa* cultivar Nipponbare rice roots and showed endophytic colonization. The phenotypic analysis showed that PsJN, Pk and Bv have respectively neutral, positive and negative effects on rice productivity. Then, the transcriptomes of leaves and roots of axenic and PsJN-colonized plants were produced to characterize the transcriptional response of rice to PsJN. Finally, we performed a comparative analysis of rice phenotypic and transcriptomic response to PsJN, Bv and Pk. The comparative transcriptomic analysis revealed a more conserved response in leaves than in roots. Also, strain-specific root transcriptomes are enriched in genes implicated in immunity, hormone signaling and secondary metabolism. Finally, surprisingly the transcriptomic responses to the two *Paraburkholderia* strains were markedly dissimilar, indeed the response of rice to PsJN shared characteristics of the response to both Pk and Bv.

## Results

### A smaller population of PsJN cells can colonize the rice roots endosphere compared to rice native *Burkholderia s.l.* endophytes

**Figure 1:**
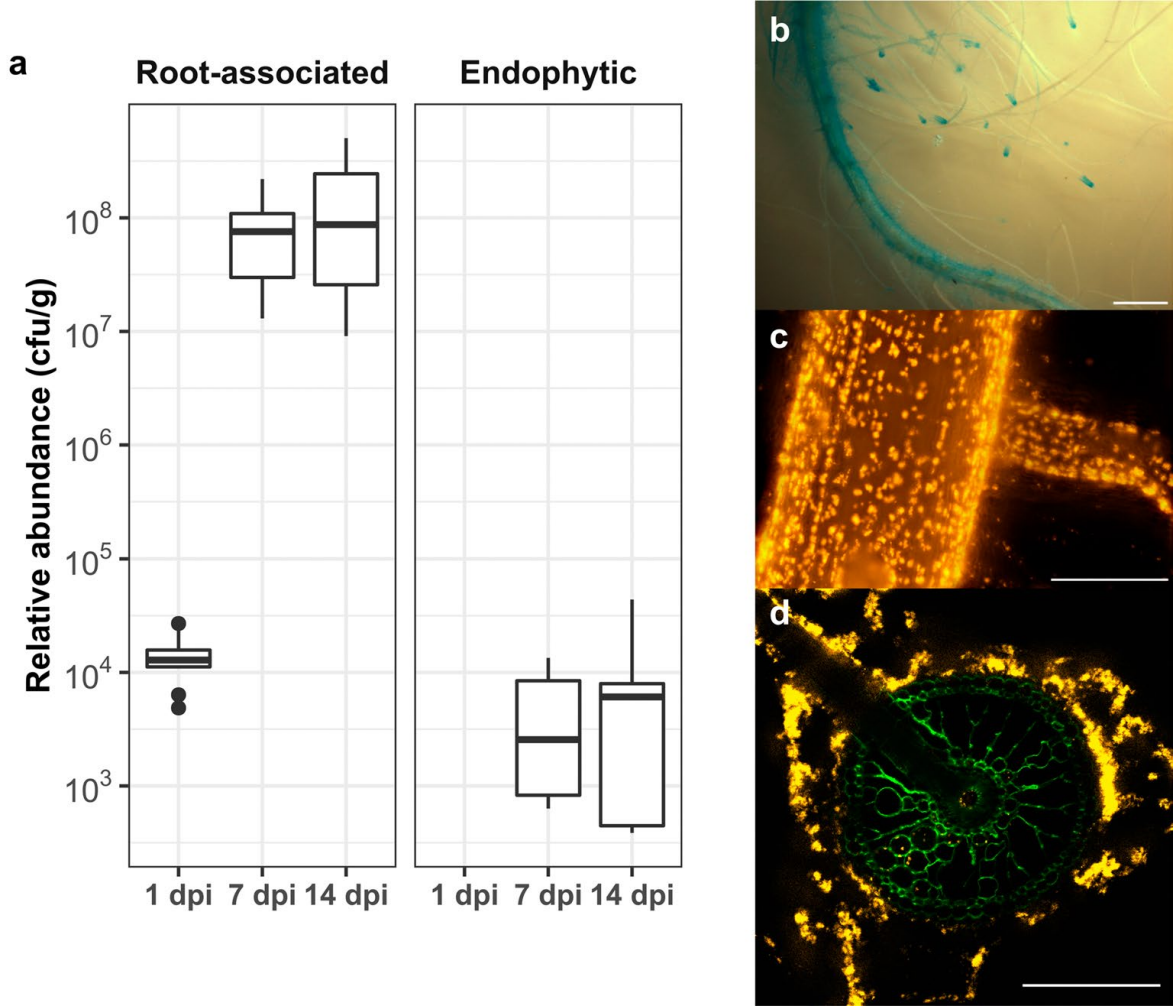
Endophytic colonization of rice roots by PsJN. (**a**) Population dynamics of DsRed-tagged PsJN associated with rice roots. The data reported are the median of bacterial population size from 9 plants for 1 dpi and 18 plants-conducted in two independent experiments- for 7 and 14 dpi. and 5 plants. For the endophytic compartment the data correspond to 5 biological replicates (**b**) Colonization of rice primary root picture by PsJN::pINGUS. White bar represents 1 mm. (**c**) Epifluorescence microscopy pictures of the colonization of rice primary roots at 7 days post-inoculation by PsJN::pIN29 cells. White bars represent 200 μm (**d**) Confocal microscopy picture of a cross-section of rice primary roots at 7 days post-inoculation by PsJN::pIN29 cells. White bars represent 200 μm.

The capacity of PsJN to colonize several plant species has been previously described, however its ability to colonize rice roots has never been established. To test this, we inoculated a DsRed-tagged strain on rice (*O. sativa* cv Nipponbare) roots of 4-days old plants grown in hydroponic conditions. PsJN colonization was first quantitatively evaluated by estimating the size of the bacterial population associated to rice roots (Fig. 1a). PsJN rapidly colonized rice roots by reaching a median value of population size of 1.28 × 10^4^ cfu g^−1^. Afterwards, from 1 to 7 dpi, the population size of PsJN massively increases to reach a median value of 6.57 × 10^7^ cfu g^−1^. Finally from 7 to 14 dpi, the bacterial population size almost doubled to reach a median value of 1.26 × 10^8^ cfu g^−1^. To estimate the amount of PsJN cells internally colonizing the rice root tissues, we conducted surface disinfection experiments. The size of the endophytic population of PsJN is dramatically (10,000 to 100,000 times) smaller than the root-associated population. Nonetheless, from 7 to 14 dpi, similarly to the total root-associated population, the endophytic population increased by a factor 1.5 by increasing from a median value of 4.07 × 10^3^ to 6.09 × 10^3^ cfu g^−1^.

We then carried histochemical and fluorescence microscopy analysis to qualitatively describe the colonization of rice roots by PsJN cells. Our results show that PsJN cells can massively colonize the surface of root hairs, the root cap of lateral roots and the surface of the primary root (Fig. 1b,c). The GUS histochemical staining experiments showed that the surface of the primary root is largely colonized, as well as the root caps of lateral roots while their surface remain relatively free of bacteria comparatively to the primary root (Fig. 1b). When observing the colonization of primary root epidermis, bacterial aggregates can be observed on the primary roots and lateral root emergences (Fig. 1c). Finally, root cross-section followed by confocal microscopy revealed that PsJN cells can colonize the inner tissues of rice roots. Indeed, fluorescent cells can be found associated to the cell wall of epidermic and cortical cells and most notably, PsJN cells have been observed in the xylem (Fig. 1d). These results show by two approaches that PsJN is able to establish endophytically in rice roots in our experimental conditions.

As PsJN was not originally isolated from rice plants, while it colonizes its endosphere in our conditions, it can be considered a non-native endophyte. We wanted to compare rice PsJN-induced phenotypic and transcriptional responses to the colonization by *Burkholderia s.l.* endophytes naturally interacting with rice. We chose *Paraburkholderia kururiensis* M130, hereafter termed *Pk*, and *Burkholderia vietnamiensis* TVV75^T^, hereafter termed *Bv*, which were isolated from rice roots and previously shown to endophytically colonize rice roots^14^. The colonization pattern of PsJN was then compared to the two model endophytes of rice Pk and Bv using the same protocol. If PsJN appears to colonize slower the root surface at 1 and 7 dpi, it reaches a similar population level than Bv and Pk at 14 dpi (Supplementary Fig. S1). However, PsJN colonized less (10 to 100 times) the root interior at 7 and 14 dpi compared to Bv and Pk. The epifluorescence microscopy pictures of a lateral root emergence colonized by Pk and Bv shows different patterns. Indeed, Pk appears to colonize more homogeneously than PsJN, with smaller micro-colonies (Supplementary Fig. S1). On the other hand, the colonization pattern of Bv is different than the *Paraburkholderia* strains, as we observed patches of cells with high fluorescence intensity and potential intracellular colonization of epidermic cells. Furthermore, the confocal microscopy pictures of root section show that xylem colonizing Pk cells can be observed (Supplementary Fig. S1). Besides, Bv endophytic colonization seems to be restricted to rice root epidermic cells.

### The transcriptional analysis of rice response to PsJN shows important and dynamic regulations of defense, JA signaling and iron homeostasis in roots

To identify differentially expressed genes (DEGs) in leaves and roots following PsJN inoculation, we used RNA-sequencing on roots and leaves at 7 dpi in a hydroponic system (see the Methods section). Total RNA was extracted from three biological replicates per treatment (control or inoculated), each replicate being a pool of 5 plants. On average 42,782,744 reads were sequenced from each sample of which 69% were uniquely mapped to the rice genome (IRGSP, version1) (Supplementary Table S1). The genes with an FDR adjusted *p*-value inferior to 0.01 were considered significantly differentially expressed. In leaves, 2646 and 2337 DEGs (Supplementary Table S2) were identified as up and down-regulated genes respectively whereas in roots 379 and 609 DEGs, respectively, were identified (Supplementary Table S3).

**Figure 2:**
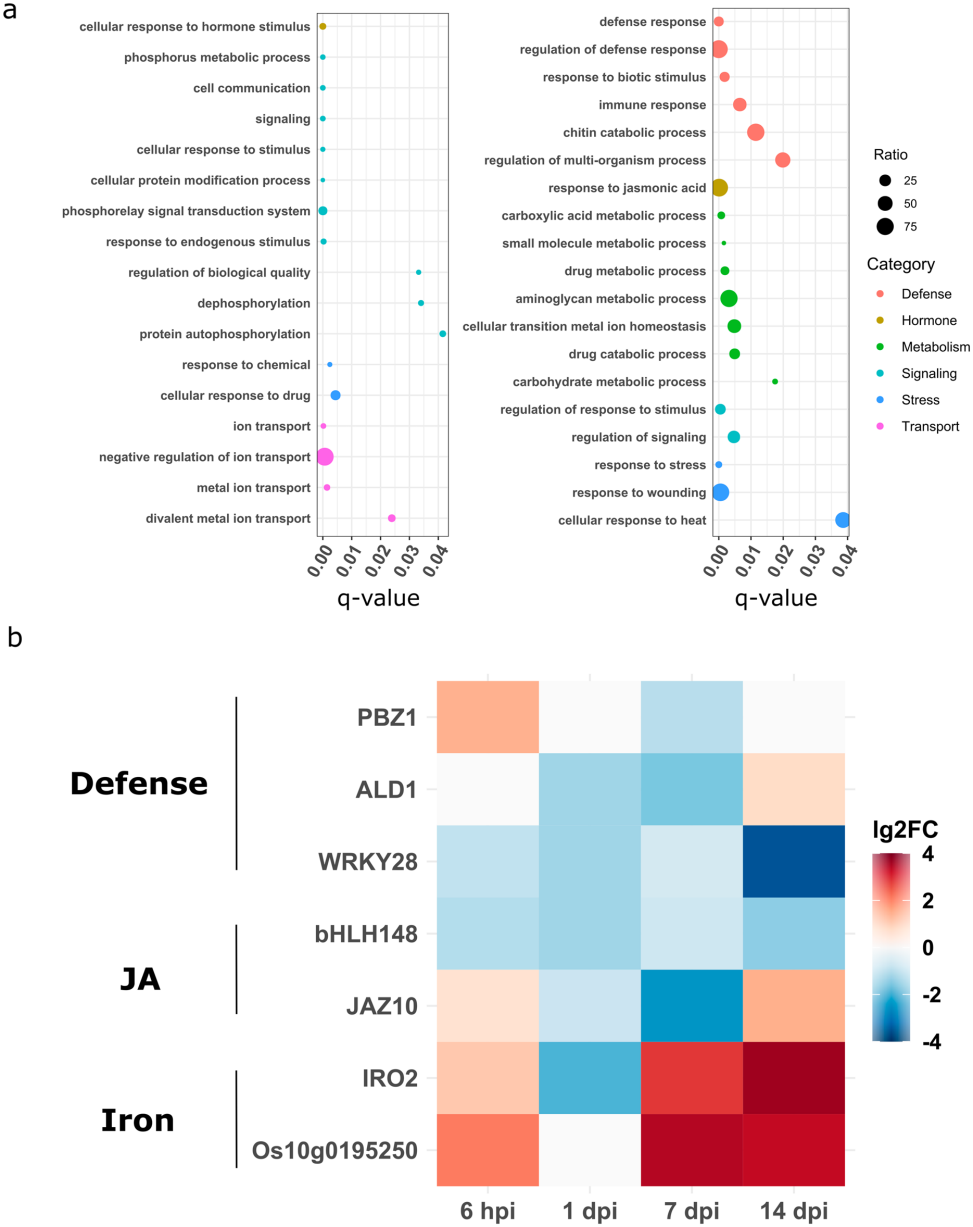
Root transcriptional response to PsJN colonization. (**a**) Enrichment analysis of Biological Process GO terms of PsJN-treated rice root transcriptome. Y-axis corresponds to the significantly enriched GO terms (FDR < 0.05 and an enrichment ratio threshold of 2), X-axis corresponds to the adjusted *p*-value, the enrichment ratio represent by the size of the dots corresponds to the ratio between the proportion of genes related to the given GO term in the transcriptome and the proportion of genes related to the given GO term in the rice genome. Left and right panels respectively correspond to up- and down-regulated DEGs (**b**) The values of mean log2FoldChange of 7 genes compared to a non-inoculated control over 6 hpi, 1, 7 and 14 dpi were represented as heatmaps. Expression level is color-coded with red indicating up-regulation and blue indicating down-regulation. Only values greater than 0.5 or lower than − 0.5 were represented.

To identify the biological processes transcriptionally regulated in rice during the interaction with PsJN, we performed GO terms and KEGG pathway enrichment analysis. Significantly enriched GO terms in root transcriptome encompasses terms related to hormone signaling, metal ion transport (especially iron) and signaling for the up-regulated DEGs and genes implicated in immunity, carbon fixation and secondary metabolism for the down-regulated genes (Fig. 2a, Supplementary Table S4). KEGG pathway enrichment analysis on down-regulated DEGs in roots also pinpoints an enrichment of defense-related genes such as *PR1*, *PBZ1* and *ALD1* as well as jasmonic acid (JA) signaling such as *JAZ* genes. In leaves, a large proportion of enriched GO terms are related to primary metabolism: carbon fixation, photosynthesis and amide metabolism are enriched among the up-regulated genes (Supplementary Fig. S2, Supplementary Table S4). For the down-regulated genes, namely, carboxylic acid metabolism and starch biosynthesis are enriched. Also, GO terms related to developmental process are significantly enriched for both up and down-regulated DEGs (Supplementary Table S4).

To get a better view of some of the genes highlighted by the RNAseq analyses, we performed a time course experiment based on RT-qPCR (qPCR) analysis of genes implicated in the process highlighted by the GO term and KEGG pathway such as defense, hormones and iron metabolism. We chose defense-related genes *PBZ1*, *ALD1* and *WRKY28* mentioned above. Also, the transcription factor *bHLH148* and *JAZ10* were added for the JA signaling pathway.

Finally, *IRO2* and *Os10g0195250* were added to exemplify the metal ion transport pathway. All these genes were analyzed in root tissues sampled at 6 h, 1-, 7- and 14-days post inoculation. The Fig. 2b shows through a heatmap the expression kinetics of all those genes. Interestingly, the expression of *PBZ1* and *ALD1* at 7 dpi confirms by qPCR the down-regulation in response to PsJN detected by RNA-Seq. Additionally, an early up-regulation of *PBZ1* was detected at 6 hpi while *ALD1* is up-regulated at 14 dpi. For the expression of JA-related genes, we demonstrated a down-regulation of *WRKY28* and *bHLH148* at all time points. Furthermore, *JAZ10* expression undergoes up-regulation at 6 hpi and 14 dpi while being down-regulated at 1 and 7 dpi. This pattern of early induction of expression at 6 hpi followed by a transient down-regulation phase prior to a strong induction of the expression can be observed for the kinetics of iron-related genes *IRO2* and *Os10g0195250*.

### Rice response to endophytic colonization by PsJN is atypic from the response to rice native *Burkholderia s.l.* endophytes

**Figure 3:**
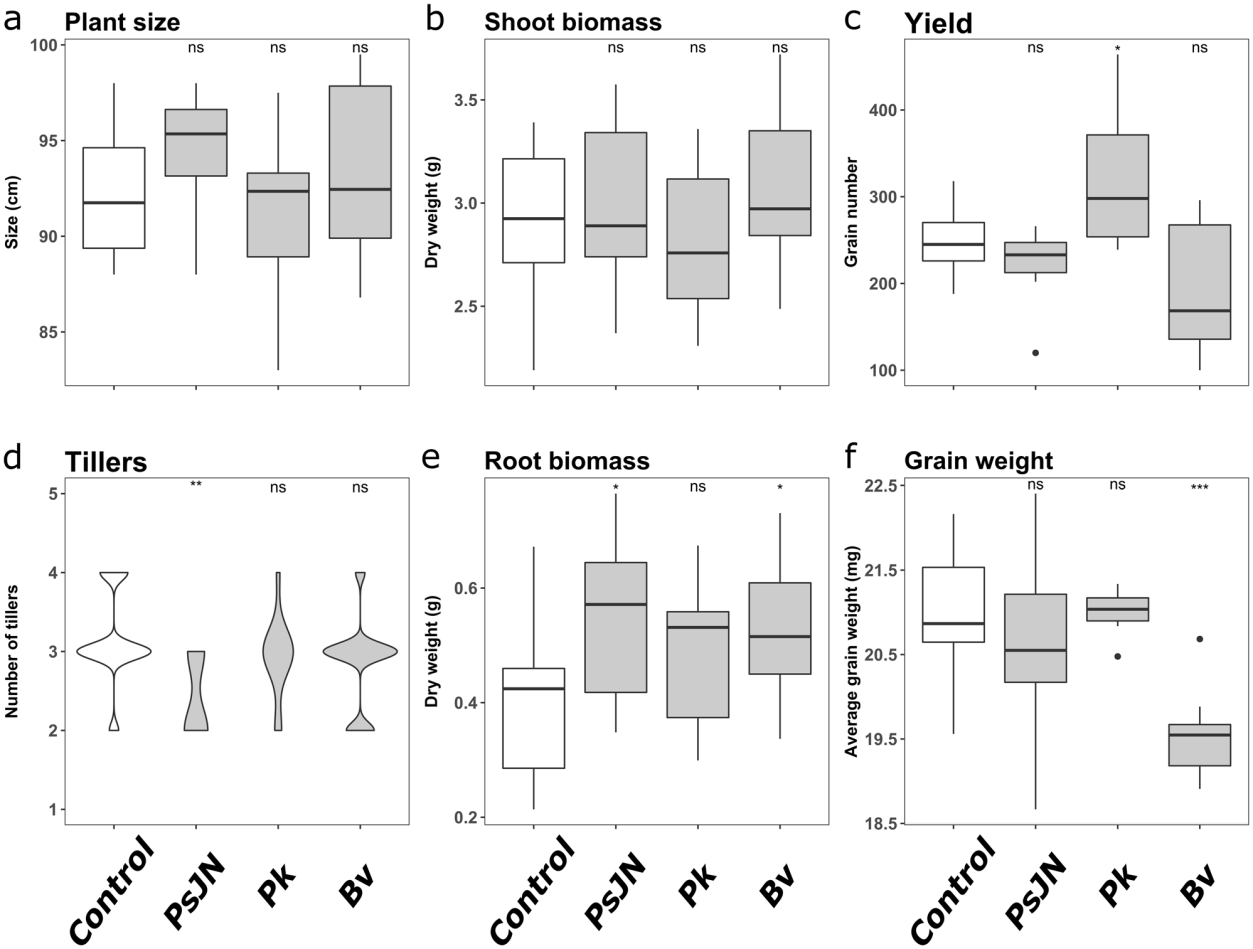
Analysis of phenotypic traits of rice in response to three *Burkholderia s.l.* endophytes. Analysis of the phenotypic response of rice to the inoculation by *P. phytofirmans* (PsJN), *P. kururiensis* (Pk) and *B. vietnamiensis* (Bv) or mock-inoculated (Control). Results presented in (**a**), (**b**), (**d**) and (**e**) correspond to the mean results obtained for 12 plants for 2 months post-inoculation with 10^8^ bacterial cells one week after sowing. Results presented in (**c**) and (**f**) correspond respectively to the number of seeds and the average grain weight produced per pot out of 8 pots, each containing 4 plants grown for 4 months post-inoculation with 10^8^ bacterial cells one week after sowing. Significant levels in variations were determined using Student’s t-test for plant size, shoot biomass and root biomass and Wilcoxon test for number of tillers, yield and grain weight (*p* < 0.05, *p* < 0.01 and *p* < 0.001 for *, ** and *** respectively).

Phenotypic analysis of soil-grown rice plants inoculated with PsJN, Bv and Pk showed no significant effect on plant size or shoot biomass (Fig. 3a,b) two months after inoculation while PsJN inoculation significantly decreased the number of tillers (Fig. 3d). On the other hand, PsJN and Bv inoculation significantly increased root biomass compared to uninoculated plants (Fig. 3e). However, Pk inoculation was the only condition where a significant increase in the number of seeds was observed (Fig. 3c) whereas Bv-inoculated plants produced significantly lighter grains (Fig. 3f). Additionally, we performed colonization assays of soil-grown rice plants inoculated with the three strains at 15 dpi (Supplementary Fig. S3). This experiment firstly showed that all strains can colonize the rhizosphere and the roots of rice plants grown in soil. In addition, we show that Bv and Pk can consistently colonize the base of the rice stem whereas PsJN was only detected in 2 out of 5 stem samples. Finally, all leaves were colonized by Bv suggesting that this strain can colonize the aboveground parts of rice plants.

**Figure 4:**
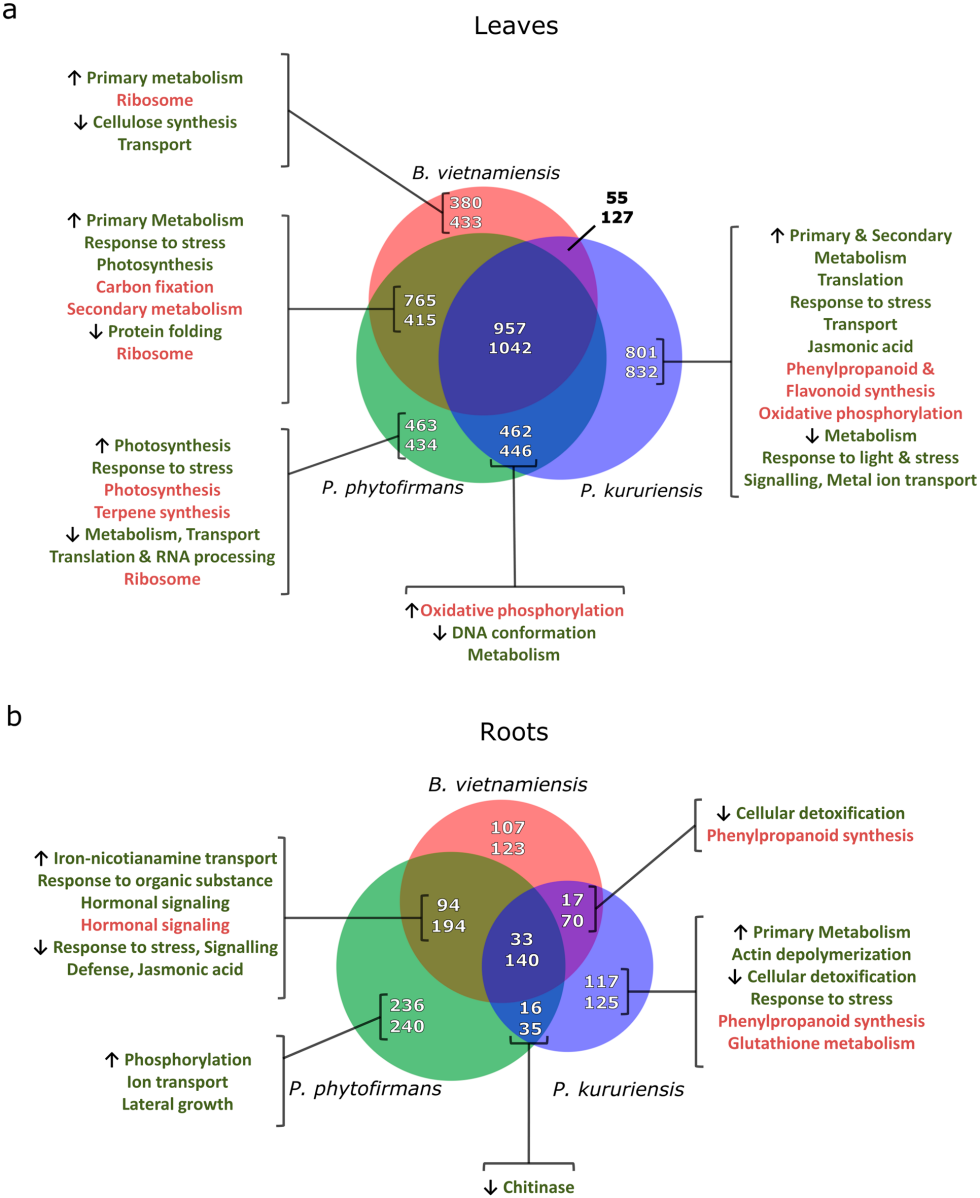
Comparative analysis of rice transcriptomic response to *Burkholderia s.l.* endophytes. Venn diagrams show the number of DEGs in response to each *Burkholderia s.l.* strains in leaves (**a**) and roots (**b**) as well as the proportion of genes shared between the responses to these three strains. For each subpart, the upper numbers correspond to the amount of up-regulated DEGs while lower numbers, the down-regulated DEGs. Representative significantly enriched GO terms and KEGG pathways related to each pool of shared or specific DEGs are respectively written in green and red.

To describe which physiological processes are commonly and specifically regulated in rice response to endophytic colonization, we performed a comparative analysis of the transcriptomic response of rice to PsJN, Pk and Bv. Additionally, the root and leaf tissues were treated and harvested simultaneously with the material used in our previous study^14^ to study respectively the local and systemic transcriptomic response to root colonization. The analysis of leaf transcriptomes of inoculated plants revealed an important conserved response of rice to three *Burkholderia s.l* endophytes*.* Indeed, 25% and 28% of up- and down-regulated DEGs respectively are commonly regulated in response to the three *Burkholderia s.l* (Fig. 4a). Additionally, among all DEGs in leaves, 56% are shared between the response to at least two endophytes. However, a very low proportion of genes (5%) are commonly regulated following Pk and Bv root colonization strictly. Finally, among all DEGs in leaves, approximately 40% are strain-specific with half of them being specifically induced by Pk. In contrast, the comparative analysis of root transcriptomes revealed a very different pattern. Indeed, among all root DEGs, 28% are specifically regulated in response to PsJN (Fig. 4b). Furthermore, in opposition to leaves, a much larger proportion of PsJN-induced DEGs in roots is shared with *Bv* than with *Pk*. Indeed, on average between up and down-regulated DEGs, 17% of DEGs are shared in response to PsJN and Bv while only 3% are shared in response to the two *Paraburkholderia* strains. Finally, a larger proportion of DEGs in common are down-regulated. For example, 14% of all down-regulated DEGs are commonly responsive to the three endophytes while only 5% for the up-regulated DEGs.

This analysis reveals an important overlap of PsJN-induced gene regulations with both Pk and Bv-induced regulations in leaves. On the other hand, most of DEGs regulated by both rice-isolated endophytes are also induced following PsJN root colonization. This corroborates with principal component analysis (PCA) of the normalized reads in the transcriptome of leaves, indeed the response to Bv overlaps with the response to PsJN (Supplementary Fig. S4a). On the other hand, PsJN root colonization specifically induced the regulation of the largest proportion of genes (Fig. 4b) leading to a separation of the response of roots to PsJN and Bv on the PCA analysis (Supplementary Fig. S4b).

### Defining the core transcriptomic response of rice to endophytic colonization

**Figure 5:**
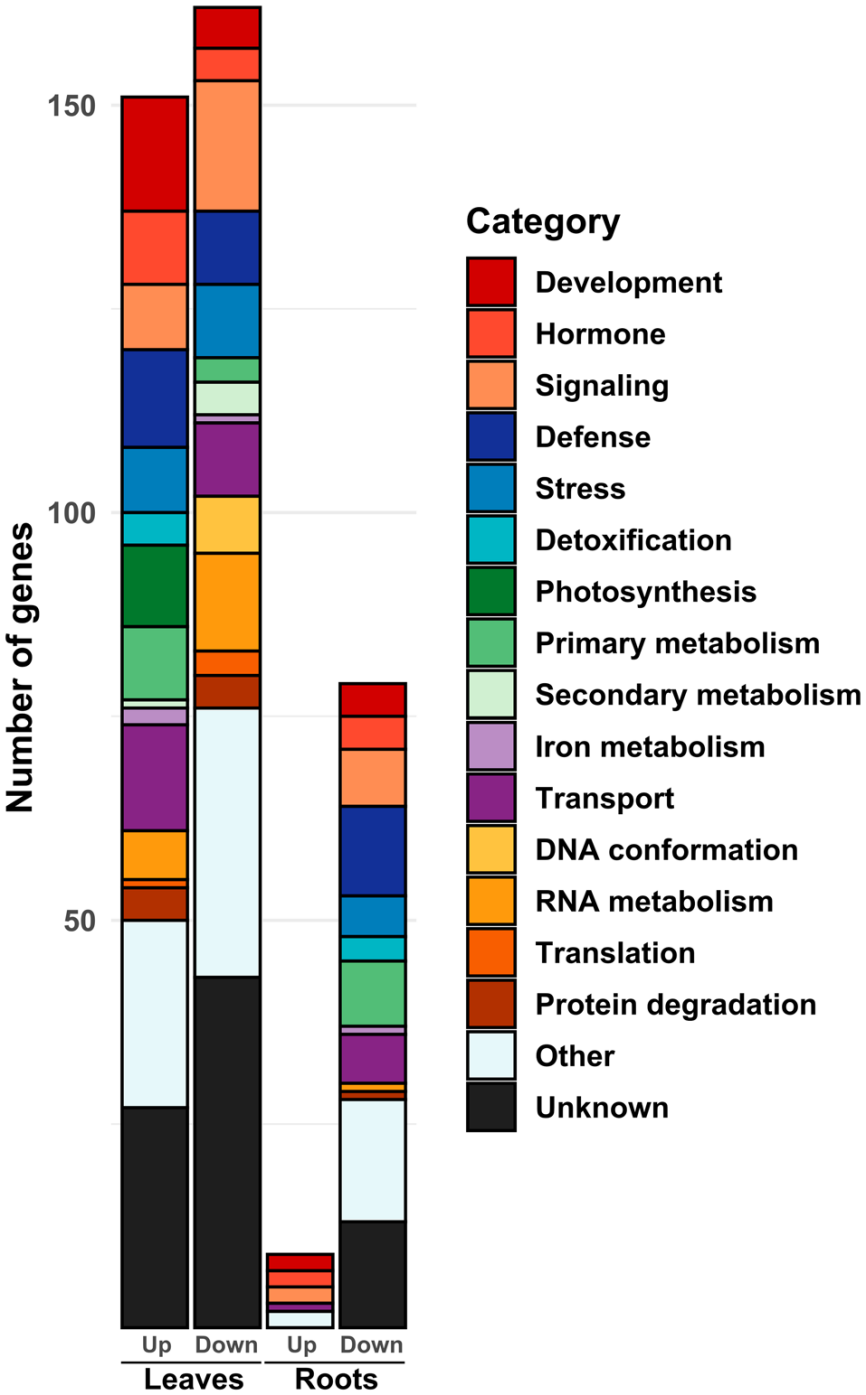
Common transcriptomic response of rice to *Burkholderia s.l.* endophytes. Number of common significantly DEGs highly regulated (|Log_2_FoldChange|> 1) in response to PsJN, Pk and Bv related to functional categories.

To further describe the physiological processes regulated in response to endophytic colonization, we performed Gene Ontology (GO) terms and Kyoto Encyclopedia of Genes and Genomes (KEGG) pathway enrichment analysis on the different pool of DEGs shared or not between the response to the three endophytic species (Fig. 4, Supplementary Tables S5, S6, S7 and S8). Following this, we conducted a manual functional categorization of the most highly regulated DEGs (|Log_2_FoldChange|> 1) for the common response to the three endophytes additionally to the enrichment analysis (Fig. 5, Supplementary Table S9).

For the commonly up-regulated DEGs in leaves, genes implicated in the response to stresses, photosynthesis and oxidant detoxification are significantly enriched (Supplementary Table S5). Among the top up-regulated DEGs we identified genes related to development, mainly genes encoding for transcription factors and cell wall-related metabolic processes, transport, mostly proton pumps (Supplementary Table S9). Also, genes related to immunity were identified among the most highly up-regulated DEGs in leaves. Both regulatory genes like *WRKY7*, *WRKY28* or *MKK4* and also antimicrobial compounds encoding genes such as *PR2*, *PAL9* and *Cht6* are commonly up-regulated in response to PsJN, Pk and Bv.

Among the down-regulated DEGs in leaves, the largest proportions correspond to genes related to signaling, mostly kinase-encoding genes, and RNA and DNA metabolism especially genes related to RNA polymerase II and chromatin conformation (Fig. 5). Furthermore, we identified DEGs related to transport, particularly transporters associated to the vacuole and related to N metabolism such as *AMT3.3* and a Trp/Tyr transporter. Finally, down-regulated defense-related DEGs are mostly putative resistance genes, with 8 out of 9 genes encoding for NB-ARC proteins (Supplementary Table S9).

As previously described, the root common response to the three endophytes is much more restricted in proportion compared to leaves. This is particularly the case of the up-regulated DEGs which also reach lower level of relative expression (Fig. 5, Supplementary Table S9). This low proportion of commonly up-regulated DEGs in roots is also associated to no significant GO term or KEGG pathway (Supplementary Table S7). However, among the most highly up-regulated genes, we identified two genes encoding for aminocyclopropane carboxylate oxidase (Os06g0573900 and Os02g0771600), two genes encoding for putative protein kinase (Os09g0551251 and Os04g0633900), two genes related to root development (*RSL9* and Os05g0552600) and a putative sugar transporter (*Os03g0197100*).

Additionally, enrichment analysis showed that the KEGG pathway "Glycolysis/Gluconeogenesis" is significantly enriched among the common down-regulated DEGs in roots (Supplementary Table S8). Also, among the most highly regulated DEGs, the second functional category in proportion is primary metabolism with genes related to carbon metabolism and most particularly four genes implicated in pyruvate metabolism (*Os05g0469600*, *Os03g0432100*, *Os06g0104900*, *Os05g0469800*) also three genes related to amino acid synthesis (*Os05g0244700*, *Os01g0720700* and *ARD3*).

However, genes related to immunity represent the largest category among the most highly down-regulated genes (Fig. 5). Particularly, we identified two genes encoding for transcription factors of the WRKY family (namely *WRKY62* and *WRKY76*), three genes implicated in secondary metabolism involved in defense response (*CCR1*, *CCR10* and *Os03g0122300*), two genes implicated in the response to SA (*RH2* and *RH3*) and finally one gene of the *PR* family (*PR1a*).

### Comparative analysis of transcriptomes reveals important strain-specific transcriptional regulation of primary and secondary metabolism

We then described the transcriptional regulations common to the response to two *Burkholderia s.l.* strains or specific to the response to each strain. In leaves, this comparative analysis revealed that the most significantly enriched processes are related to energy production and primary metabolism. Particularly, the common transcriptomic response to PsJN and Bv is significantly enriched in genes related to carbon fixation, photosynthesis and carboxylic acid metabolism (Fig. 4a and Supplementary Table S5). On the other hand, the common response to PsJN and Pk is enriched in genes related to oxidative phosphorylation according to both GO terms and KEGG pathways. Additionally, the strain-specific response to PsJN and Pk showed an enrichment in photosynthesis and respiration respectively.

Furthermore, KEGG enrichment analysis showed strain-specific transcriptional regulations of secondary metabolites pathways. For example, the specific response to Pk in leaves, is enriched in genes implicated in the phenylpropanoid and flavonoid synthesis pathways (Fig. 4a). Contrastingly, in response to PsJN, genes implicated in the terpene synthesis are specifically enriched among up-regulated DEGs. No specific pathway is enriched among the common DEGs in response to PsJN and Bv, however the "secondary metabolism" KEGG term is significantly enriched. Similarly, the root transcriptomic response to Pk, both specific and shared with Bv is enriched for the down-regulation of genes implicated in phenylpropanoid synthesis (Fig. 4b and Supplementary Table S8).

**Figure 6:**
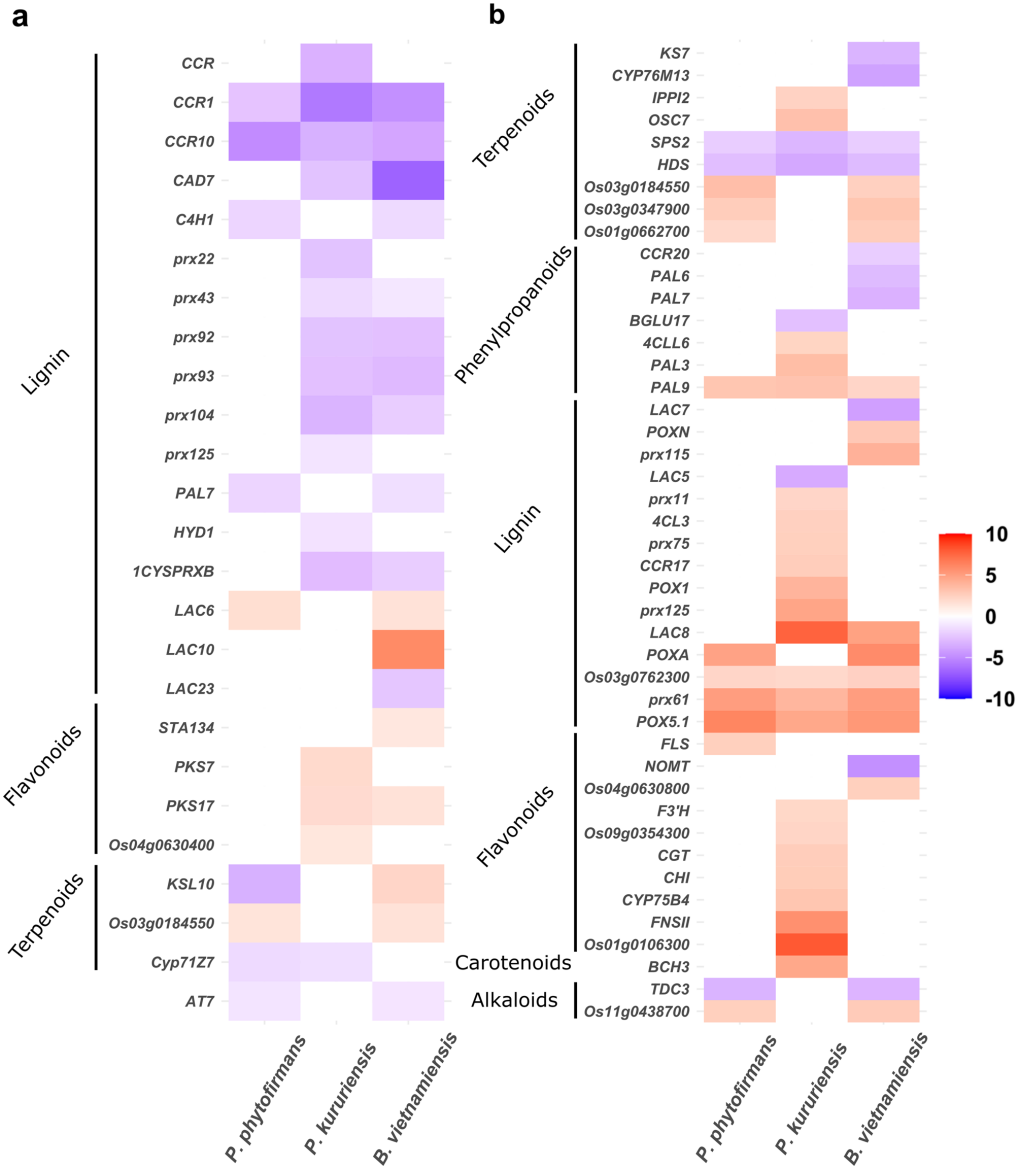
Comparative analysis of secondary metabolites genes expression at 7 dpi in rice roots and leaves in response to *Burkholderia s.l.* endophytes. Relative expression of DEGs (|Log_2_FoldChange|> 1) related to secondary metabolism in rice roots (**a**) and leaves (**b**) in response to the colonization by *P. phytofirmans*, *P. kururiensis*, *B. vietnamiensis* at 7 days post-inoculation. Expression level is color-coded with red indicating up-regulation and blue indicating down-regulation.

**Figure 7:**
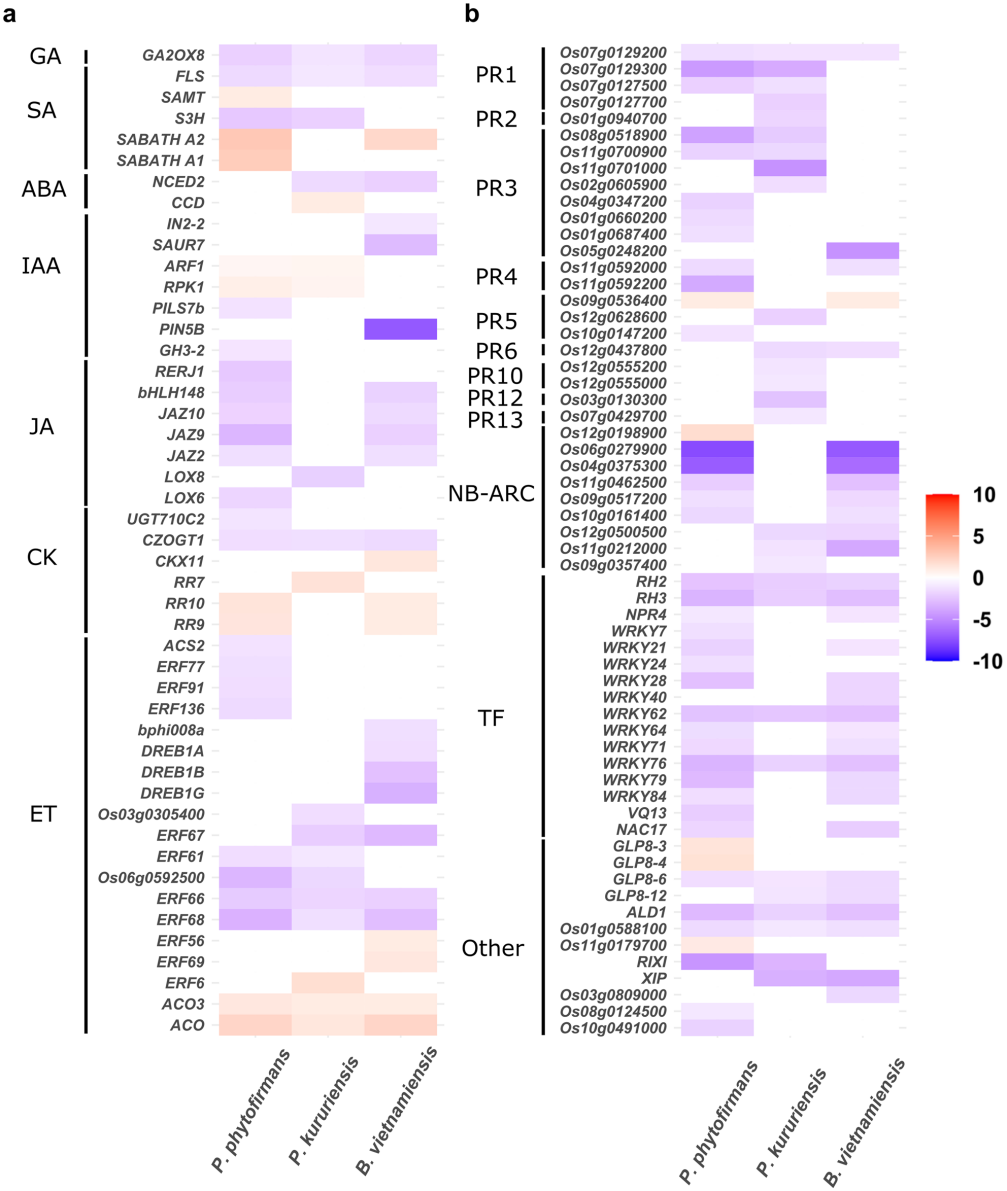
Comparative analysis of hormone and immunity-related genes expression in rice roots in response to *Burkholderia s.l.* endophytes. Relative expression of (**a**) hormone-related and (**b**) immunity-related DEGs (|Log_2_FoldChange|> 1) in rice roots in response to the colonization by *P. phytofirmans*, *P. kururiensis*, *B. vietnamiensis* at 7 days post-inoculation. Expression level is color-coded with red indicating up-regulation and blue indicating down-regulation.

This global functional analysis of rice transcriptomic response to PsJN, Pk and Bv led us to focus on the differential expression of genes implicated in secondary metabolism, hormonal signaling and defense in response to the three endophytes. To further deepen our analysis, we compared the expression of the most-highly regulated DEGs (|Log2FoldChange|> 1) implicated in these processes during the interaction with PsJN, Pk and Bv (Figs. 6 and 7).

Relatively few genes implicated in secondary metabolism are commonly regulated following endophytic colonization, namely two backbone terpene synthesis are down-regulated (*HDS* and *SPS2*), and 4 genes related to phenylpropanoid synthesis are regulated (*POX5.1*, *prx61*, *PAL9* and Os03g0762300) mostly specifically implicated in lignin synthesis except for *PAL9* (Fig. 6b and Supplementary Table S10). Furthermore, genes implicated in lignin synthesis appear to be globally down-regulated in roots while almost all genes related to lignin synthesis in leaves are up-regulated in response to root colonization.

Particularly, Pk-inoculated plants induced the regulation of 10 and 12 genes implicated in lignin synthesis, respectively up-regulated in leaves and down-regulated in roots (Fig. 6a). Consistently with the GO term enrichment analysis, rice response to Pk stands out because of the specific up-regulation of genes related to flavonoids synthesis in both leaves and roots. Inversely, PsJN and Bv colonization induced the up-regulation of 3 genes implicated in terpenoids synthesis (Os03g0184550, Os03g0347900 and Os01g0662700) and 2 genes related to alkaloids synthesis (*TDC3* and Os11g0438700) in leaves. Also, the colonization of PsJN and Bv induced in roots the regulation of respectively 4 and 3 genes related to terpenoids synthesis while only 1 is down-regulated in response to Pk.

### Immunity and hormone-related transcriptional regulations in roots reveals a largely shared response to PsJN and Bv dissimilar of the response to Pk

In roots, the transcriptomic response to the three endophytes is enriched in genes related to defense, stress response and hormonal signaling (Fig. 4, Supplementary Tables S7 and S8). Also, the common response to Bv and PsJN is enriched in genes related to hormone signaling, response to stress, signaling and defense. This led us to focus on the regulation of genes related to hormonal signaling (Fig. 7a) and immunity (Fig. 7b) to pinpoint fundamental differences in the response to colonization by PsJN, Pk and Bv.

As previously described, the common response to the three endophytes is characterized by the up-regulation of 2 *ACO* genes, implicated in ethylene synthesis. Additionally, 13 genes implicated in the ethylene signaling pathways, mostly from the *ERF* family, are down-regulated by at least one of the three *Burkholderia s.l.* strains (Fig. 7a). Notably, only Bv colonization induces the down-regulation of *DREB* genes. Also, the up-regulation of *RR* genes, implicated in cytokinin signaling is common to the response to the three endophytes although different genes are triggered by each strain. While the transcriptional regulation of genes implicated in the ethylene and cytokinin signaling appears to be shared among all conditions, only two strains trigger a substantial down-regulation of genes related to JA signaling. Indeed, as revealed by the GO term enrichment analysis, roots colonized by PsJN and Bv commonly induce the down-regulation of JA-related genes: *JAZ2*, *JAZ9*, *JAZ10* and *bHLH148* (Fig. 7a). Additionally, *LOX6* and *RERJ1* are specifically down-regulated by PsJN.

Finally, the regulation of defense-related genes probably reveals the most striking differences in the local response to the three endophytes (Fig. 7b). Indeed, while Pk root colonization triggers only the down-regulation of two *WRKY* genes, 8 additional genes from this family are down-regulated in response to both PsJN and Bv. Furthermore, 5 genes encoding for putative resistance proteins (NB-ARC) are among the most down-regulated genes following PsJN and Bv colonization specifically. On the other hand, the response of rice to Pk is characterized by the down-regulation of 15 Pathogenesis-Related (*PRs*) genes. Although, PsJN colonization also induces the down-regulation of chitinase-encoding genes (*PR3* family), the regulation of genes from the *PR10*, *PR12* and *PR13* families is specific to the response to Pk (Fig. 7b).

## Discussion

### PsJN can colonize the rice root endosphere while not impacting rice yield in contrast to rice-native endophytes

Unraveling the molecular bases of plants response to various endophytes will help identify the physiological processes implicated in the establishment of plant-endophyte interactions. Based on a previous study analyzing the transcriptional response of rice to two rice-isolated *Burkholderia s.l.* endophytic strains^14^, we performed a comparative analysis of the phenotypic and transcriptomic responses of *Oryza sativa*, cultivar Nipponbare, to *Paraburkholderia phytofirmans* PsJN, a wide spectrum endophyte, with the response to rice-native *Burkholderia s.l.* endophytes.

The analysis of rice root colonization showed that PsJN can colonize the endosphere of rice roots. Indeed, from 7 to 14 dpi the amount of PsJN cells colonizing rice roots reaches approximately 10^8^ cfu g^−1^ which is consistent with the level of colonization observed for several other plant-rhizobacteria models^16^. On the other hand, this population size of PsJN cells is significantly smaller than for rice-native *Burkholderia s.l.* endophytes *Pk* and *Bv*^14^.

(Supplementary Fig. S1b). This is particularly the case for the early time point at 1 and 7 dpi for the root-associated population and for the endophytic population at both time points when compared to Pk.

Although an important colonization of rice roots by PsJN cells was observed, the different root areas are not homogeneously colonized. Indeed, PsJN cells particularly accumulate in the root hair zone and on lateral root tips (Fig. 1b). This was also observed during the colonization of grapevine by PsJN^17^. Interestingly, these root areas correspond to the most nutrient-rich zones. Particularly, the root hair zone is a hotspot in terms of exudates concentration^18^. Also, the root cap cells could be microbial hotspot for several reasons. First, these cells exude large amount of polysaccharide-based mucilage which could be used as nutrients by PsJN cells, as it was previously observed in pea^19^. Secondly, root cap cells have a shorter life cycle compared to other root cells^20^, which is a consequence of a programmed cell death. This leads to the leakage of the cellular content of which some components could be used as nutrients by bacteria. Finally, PsJN cells were also observed colonizing the cortex and the xylem of rice roots similarly to Pk (Supplementary Fig. S1a) and previous observations particularly for grapevine plantlets^21,22^.

The analysis of the phenotypic response of rice plants to the inoculation with PsJN, Pk and Bv revealed no significant increase of the aboveground biomass contrarily to what was previously described in response to Pk and Bv^15,23^. This can be related to the growth conditions we used for the phenotypic assay especially in terms of nutrients available in the substrate. However, we measured a significant increase of the number of grains produced by plants inoculated with Pk as previously described^15^. Oppositely, in our experiment plants colonized by Bv produce grains of a lower average weight. This negative impact of Bv colonization on grain weight can be related to the more invasive colonization patterns we previously described in hydroponic conditions^14^. This could be related to a reduced fitness, indeed grain size is correlated with higher germination rate and seedling vigour in rice^24^. Nonetheless, the inoculation of Bv induced a significant increase of rice root biomass. This is also the case for plants inoculated with PsJN who show an increased root biomass and a grain production like the uninoculated plants although PsJN-treated plants had less tillers (Fig. 3). In conclusion, these results suggest that in our growth conditions, the endophytic colonization of PsJN, Pk and Bv respectively have a neutral, beneficial and negative impact on rice fitness.

### Comparative rice transcriptomic response to PsJN and rice-native *Burkholderia s.l.* reveals a common response enriched in defense, hormonal and signaling regulation

The analysis of rice transcriptomic response to PsJN revealed an important enrichment in genes related to photosynthesis in leaves (Supplementary Fig. S2, Supplementary Table S4). Coherently, PsJN inoculation can induce an increase photosynthetic efficiency in *Arabidopsis*^25^ and switchgrass^26^. Also, genes related to development and reproduction are significantly enriched among the DEGs in rice leaves following PsJN inoculation similarly to what was observed in *Arabidopsis*^5^. In roots, PsJN induced the regulation of genes related to hormones previously described to be modulated during root colonization by rhizobacteria namely cytokinin^13^ and jasmonic acid^27,28^. We confirmed these RNASeq results by qPCR analyses demonstrating the maintained and transient down-regulation expression of JA-responsive genes such as *bHLH148*^29^, *WRKY28*^30,31^ and *JAZ10*^32^ respectively. Also, a number of genes related to metal ion transport are up-regulated in response to PsJN colonization similarly to what was observed in response to *Herbasprillum seropedicae*^33^. Furthermore, the rice gene Os10g0195250 was commonly induced by *Azospirillum lipoferum* and *Azospirillum sp. B510* inoculation^13^ and iron deficiency^34^. We could show by qPCR that this gene and *IRO2* were particularly highly over-expressed at later time points of the kinetic analysis while being induced as well at 6 hpi. This suggests that iron deficiency response is triggered in roots colonized by diverse rhizobacteria.

The most striking observation following the comparative analysis of rice transcriptome in response to PsJN, Pk and Bv is that a more conserved transcriptional response is triggered in leaves than in roots (Fig. 4). As relatively few studies analyzed the response of both roots and leaves in response to endophytic colonization, the representativity of this observation must be tempered. We can hypothesize that regulations occurring in roots are more specific in roots to trigger the appropriate response to each strain while transcriptomic regulations occurring in leaves are more related to metabolism and therefore more shared. The enrichment analysis performed on leaves transcriptomic data supports this hypothesis. Indeed, a large proportion of enriched terms are related to primary metabolism (Fig. 4a, Supplementary Tables S5 and S6). However, different energy-producing pathways appear to be triggered by Bv and PsJN on one side and Pk on the other. Indeed, while Pk inoculated plants up-regulate genes implicated in respiration, the shared response to Bv and PsJN is enriched in genes related to photosynthesis and carbon fixation. This can be one of the factors explaining the increased root biomass in response to PsJN and Bv (Fig. 3e).

However, the up-regulation of genes implicated to photosynthesis, primary metabolism and development are also part of the core transcriptomic response of rice to the three endophytic strains (Fig. 5, Supplementary Table S9). Also, a significant amount of genes regulated in leaves are related to immunity such as *WRKY7*^35^, *WRKY28*^30^ and the gene *MKK4*, which encodes for a MAPK kinase implicated in rice immunity^36^, is also commonly up-regulated in the leaves of inoculated plants. In addition, the fact that 9 putative resistance genes and 24 putative protein kinases are transcriptionally regulated may result in a reduced or enhanced susceptibility to leaf pathogens (Supplementary Table S9). Furthermore, a substantial proportion of the genes commonly down-regulated in leaves are implicated in DNA conformation and the RNA polII machinery implicated in gene silencing (Fig. 5, Supplementary Table S9). This supports the fact that chromatin compaction and post-transcriptional regulations are also implicated in the common response of plants to endophytes as previously described^37^.

In roots, two genes encoding for ACC oxidase are commonly up-regulated, this enzyme is the last one implicated in the synthesis of ethylene^38^. This was also observed in the roots of PsJN-treated *Arabidopsis* plants^4^. This could be related to the fact that all strains harbor the gene encoding for ACC deaminase which is a well-known plant growth promoting trait. Indeed, this enzyme is able to cleave ACC, an ethylene precursor, and therefore lowers plants stress response and maintains growth^39^. Although ACC deaminase has a much lower affinity towards ACC than ACO^40^, here the microbial population is very high. Therefore, the up-regulation of two ACO-encoding genes could be a way for rice roots to compensate the accumulation of microbial ACC deaminase which possibly led to the down-regulation of the *ERF* genes (Fig. 7a, Supplementary Table S11). The common response to three endophytes in roots is also characterized by the regulation of genes implicated in signaling, especially membrane receptors such as the gene *SHR5* of which an ortholog in sugarcane was shown to be down-regulated in response to endophytic colonization^41^. Also, other putative protein kinase such as *WAK21*, Os03g0643250, Os07g0251900, Os07g0668500, Os09g0551251, Os04g0633900 as commonly regulated by the three endophytes and could therefore be promising targets for functional assays.

### The strain-specific transcriptomic regulations are characterized by pool of genes implicated in secondary metabolism

Following the analysis of the common transcriptomic response of rice to three *Burkholderia s.l.* endophytes, we performed enrichment analysis to identify physiological processes differentially regulated in response to the three strains. Among the pool of genes responsive to one or two strains, most important enriched terms are related to metabolism as describe earlier but also secondary metabolism as revealed by the KEGG pathways enrichment analysis in both roots and leaves. Similarly, strain-dependent regulation of secondary metabolism were previously described in *Brassica oleracea* in response *Paraburkholderia* species^42^ and rice in response to *Azospirillum* strains^43^. Particularly, while all strain triggered the up-regulation of 3 genes related to lignin synthesis, Pk inoculation specifically up-regulates additional genes related to lignin and flavonoid synthesis (Fig. 6, Supplementary Table S10). Among the latest, *FNSII* and *CYP75B4*, are genes up-regulated in the leaves of Pk-inoculated plants, implicated in the polymerization of lignin in grasses through tricin biosynthesis^44,45^. Additionally, genes implicated in anthocyanin synthesis such as *CHI* and *F3’H*^46^ are-upregulated in leaves following Pk colonization. This may induce an increased tolerance of plants colonized by Pk to leaf pathogens.

In roots, both Pk and Bv induced the down-regulation of a number of genes encoding for peroxidases implicated in lignin synthesis (Fig. 6a). The regulation of such genes in response to the colonization by endophytes has been described in several studies^47^. Additionally, two genes encoding for Cinnamoyl-CoA reductase, namely *CCR1* and *CCR10*, are down-regulated in roots colonized by each strain (Fig. 6a). Interestingly, *CCR1* participates in root immunity through lignin synthesis^48^. Therefore, we can hypothesize that this down-regulation of genes implicated in lignin synthesis in roots is related to suppression of root immunity to accommodate bacterial endophytes. Oppositely, the fact that plants colonized by endophytic fungi trigger the up-regulation of genes implicated in lignin synthesis led to propose that it was a local response to infection to fungal hyphae whether pathogenic or not^49,50^.

### Defense-related transcriptional regulations triggered in rice by PsJN endophytic colonization are atypical from the response to the rice-native Pk and Bv endophytes

To colonize rice roots, bacterial cells need to evade plant immunity. Indeed, the perception of conserved microbe-associated molecular patterns (MAMPs) by plant cells usually leads to the activation of MAMPs-triggered immunity (MTI)^51^. This basal immune response is mainly characterized by the production of Pathogenesis-Related (PR) proteins during microbial colonization of plant tissues. Beneficial and pathogenic microbes have evolved several mechanisms to avoid or suppress this response^52,53^. Therefore, all three endophytes used in this study must dynamically suppress rice root immunity to colonize the tissues. We could show by qPCR (Fig. 2b) that *PBZ1* and *ALD1*^54^ are down-regulated at 7 dpi by PsJN while being respectively up-regulated at 6 hpi and 14 dpi. Interestingly, important variation of expression of *PBZ1* and SA-related genes, between different time points post inoculation, were previously described in rice roots inoculated with *Pseudomonas putida* RRF3^55^. Additionally, the functional analysis of the RNA-Seq data revealed that most of the defense-related genes are down-regulated in response to the three *Burkholderia s.l.* endophytes (Fig. 7b). Interestingly, by comparing the response to three endophytes, two patterns emerge. On one hand, the inoculation of Pk induced the down-regulation of genes from a number of PR family. On the other hand, PsJN and Bv induced the down-regulation of several *WRKY* genes, NB-ARC genes and also induced the down-regulation of several JA-related genes (Fig. 7a). Interestingly, several examples of beneficial microbes suppressing root immunity depending on JA signaling components have been described^56^. For example, JA-deficient rice plants were more susceptible to the colonization by the bacterial endophyte *Azoarcus olearius* BH72^57^. Consequently, the analysis of the role of JA in the establishment of the interaction between the three *Burkholderia s.l.* endophytes and rice could be very interesting to investigate. Regarding these results we could hypothesize that the Pk-induced suppression of root immunity in a JA-independent manner in opposition to the response to PsJN and Bv. The similarities between the responses to PsJN and Bv suggest that the belonging to the genus *Paraburkholderia* or *Burkholderia s.s.* or the plant of isolation does not drive the mechanism by which the host immune response is suppressed. The characterization of the transcriptomic response of rice to a wider diversity of *Burkholderia s.l.* would need to be performed to further deepen this hypothesis. In addition, investigating the role of bacterial proteins and metabolites on the response of the host plant would be particularly interesting. Indeed, we have previously identified several pathways such as Entner-Doudoroff pathway, synthesis of Vitamin B12 and the c-di-GMP signalling to be differentially involved in the colonization of rice roots by Pk and Bv ^58^. We also identified the putrescine synthesis pathway to be differentially transcriptionally regulated between rice isolated *Burkholderia s.s.* strains in response to rice root exudates^59^. It would be particularly interesting to investigate the response of rice plants to mutant strains lacking such component to relate bacterial functions to host response.

## Methods

### Plants and bacterial cultivation

*Oryza sativa L. ssp. japonica* cv Nipponbare was used in this study. For all experiments, seeds were dehusked and sterilized as previously described^14^. Briefly, seeds were sterilized with 70% EtOH and 9.6% NaClO supplemented with 1% Tween 20, then rinsed with sterile distilled water and a 2% thiosulfate solution. Sterilized seeds germinated in sterile distilled water for 24 h and on H_2_O agar plate for 30 h at 28 °C. Homogeneously germinated seeds were finally transferred to sterile magenta boxes (SPL Lifesciences Co. Ltd) holding autoclaved perlite and 200 mL of sterile hydroponic medium. Plants were grown in a growth chamber (16 h light; 8 h dark; 28 °C; 70% humidity).

*Paraburkholderia phytofirmans* PsJN^T^ was cultured as follows: Glycerol stocks (20%) of bacterial cells conserved at − 80 °C were plated on low salt LB (Sigma-Aldrich) agar plates and incubated for 72 h at 28 °C. 20 mL Liquid LB low salt medium were then inoculated 16 h under agitation (180 rpm) at 28 °C. 500 µL of overnight culture were inoculated in fresh liquid medium for 2 h. Bacterial cells were then centrifuged for 5 min at 4000 rpm and resuspended in sterile distilled water. Each plantlet was inoculated with 10^7^ bacterial cells 4 days after sowing in hydroponic system. For greenhouse experiments (28 °C, 60% humidity), homogeneously germinated seeds were inoculated with 10^7^ bacterial cells when transferred in round small pots (Ø = 5 cm, h = 7 cm). At 15 dpi, homogeneously grown plantlets were transferred to 12 × 12 × 18.7 cm pots (1 per pot). The substrate used was the GO M2 growing media (Jiffy).

### Bacterial transformation

*Paraburkholderia phytofirmans* PsJN^T^ cells were transformed by electroporation with the pIN29 plasmid^60^ and its derivative pINGUS (Supplementary Fig. 3). The latest was obtained by replacing the gene encoding for the DsRed fluorescent protein by the *gusA* gene from the GusSH vector^61^ which encodes for a β‐glucuronidase. The primers used to amplify the gusA gene were: Fwd:5’GACCGCAAGATCTCTCCTCTAT-3’; Rev:5’CGAGCGGCCGCTATCGAATTAA-3’). A BglII + NotI double digestion (NEB) was used to replace the DsRed fragment with the *gusA* gene which was directionally inserted using a T4 DNA ligase (Promega). Both plasmids contain a chloramphenicol resistance gene as well as the DsRed or *gusA* genes under the control of a constitutive TAC promoter. After 24 h of incubation on selective medium LBm Cm (200 μg mL^−1^) at 28 °C, the most fluorescent colonies were selected (for DsRed constructs). For GusA constructs, potential transformants were plated on LBm plates supplemented with 60 µg mL^−1^ X-Gluc and 200 μg mL^−1^ chloramphenicol. Colonies showing deep blue coloration after 24 h of incubation at 28 °C were selected for further procedures.

### Rice root colonization assays

The roots of PsJN::pIN29-inoculated plants grown in hydroponics were harvested at 1, 7 and 14 dpi. For global colonization, the roots were weighted and grinded in sterile water with a sterile ceramic bead using a FastPrep-24™ 5G at 6 m s^−1^ for 40 s. The solution was then diluted and inoculated on low salt LB (Sigma-Aldrich) selective medium containing 200 μg mL^−1^ of chloramphenicol and incubated at 28 °C for 24 h. Colony forming units (cfu) were then enumerated. In order to measure the size of the endophytic population, we followed the protocol described in King et al.^14^. The inoculated rice roots were surface-disinfected for 1 min using a solution of 1% Chloramine T (Sigma-Aldrich) supplemented with 0.1% Tween 20 and finally rinsed 4 times with sterile water. Controls of disinfection were performed by plating rinsing water on TSA medium (Sigma-Aldrich) overnight. Surface-disinfected roots were then treated as described above. The solution was then diluted and inoculated on low salt LB selective medium containing 200 μg mL^−1^ of chloramphenicol and incubated at 28 °C for 24 h.

### Visualization of root-colonizing bacteria

All microscopic observations of the bacterial colonization were restricted to the primary root to compare the colonization patterns on roots which have been in contact with the bacterial population for the same amount of time. Primary roots were harvested at 7 dpi and mounted between slide and slips cover and directly examined with an Epifluorescence Nikon Eclipse Ni-E microscope. For detection of endophytical behavior, primary roots were harvested at 14 dpi and included in 5.5% agar blocks. Cross sections of 90 µm thickness were obtained using a Microm HM 650 V vibrating blade microtome (Thermo Scientific), mounted between slide and cover slip and directly observed with a LSM 880 upright confocal multiphoton microscope (Zeiss). Histochemical localization of β‐glucuronidase activity was performed by vacuum-infiltrating a GUS staining solution containing 50 µg mL^−1^ X-Gluc (Duchefa Biochemie) for 20 min in mock and PsJN::pINGUS-treated root systems in separate containers. The containers were then incubated overnight at 37 °C, then upon blue coloration visualization, roots systems were rinsed three times using a sterile phosphate buffer solution (AMRESCO). Roots were then visualized using a Nikon SMZ1500 stereomicroscope.

### RNA extraction

For the analysis of roots and leaves transcriptional profiles, plants’ tissues were harvested in triplicate at 7 dpi. Roots and leaves of untreated plants were collected at the same time. Each biological replicate consisted of five pooled root system, or 5 pooled last mature leaves harvested from a single hydroponic system. After harvest, samples were snap-frozen in liquid nitrogen and stored at − 80 °C.

Rice roots were homogenized in liquid nitrogen using cooled mortar and pestles. Rice leaves were grinded using a TissueLyser II (Retsch) set to 30 Hz for 30 s. Total RNA extraction using TRI-reagent (Sigma) was performed according to manufacturer’s instructions. All samples were treated with DNase I (Ambion) and purified using the RNA Clean & Concentrator kit (Zymo) according to manufacturer’s instructions. The integrity and quality of the total RNA was confirmed using a Nanodrop™ 1000 spectrophotometer (ThermoFisher) and a 2100 BioAnalyzer (Agilent).

### RNA sequencing and mapping of reads

Quality of RNA was checked by determining the RNA Integrity Number (RIN) with a Fragment Analyzer (Agilent). For the library preparation, samples with a RIN value > 6 were used. 12 RNA libraries were prepared using an Illumina TruSeq stranded mRNA sample preparation kit by MGX-Montpellier GenomiX core facility (MGX) France (https://www.mgx.cnrs.fr/). Library construction, sequencing and quality assessment was performed as described in King et al*.*^14^ on an Illumina HiSeq 2500. RNA-Seq reads were aligned to the IRGSP 1.0 version of the rice genome using HISAT2 v2.0.5.1^62^. The number of reads mapped to each gene locus was counted using HTSEq-count v0.6.0^63^.

### Differential gene expression and gene ontology (GO) term enrichment analysis

DESeq2 v3.7^64^ was used to calculate differential gene expression between non-inoculated and inoculated conditions. All genes having an adjusted *p*-value inferior to 0.01 were considered as significantly differentially expressed. All functional enrichment analysis was performed using g:Profiler (version e95_eg42_p13_f6e58b9) with g:SCS multiple testing correction method applying significance threshold of 0.01^65,66^. To avoid redundancy between GO terms, we used ReviGO^67^ to reduce the number of enriched GO terms as well as replacing terms related to the exact same DEGs lists by the most representative and understandable semantic expression.

### Reverse transcription and qPCR

cDNA was synthesized from total RNA using Oligo dT (Invitrogen) and the SuperScript III Reverse Transcriptase (Invitrogen) according to manufacturer instructions. Gene expression measurements were performed using Takyon SYBR 2X qPCR Mastermix Blue (Eurogentec). The gene EF-1α was used as a reference gene to normalize expression results using the ΔΔCt method^68^ with non-inoculated samples as experimental controls. Primers used in this study can be found in Supplementary Table S12.

### Ethical approval

The seeds and the plant materials of *Oryza sativa L. ssp. japonica* cv Nipponbare were obtained from the International Rice Research Institute (IRRI) and propagated in our laboratory. All plant experiments involved in this study were carried out in accordance with relevant regulations and guidelines.

## Supplementary Information

Supplementary Figure S1.

Supplementary Figure S2.

Supplementary Figure S3.

Supplementary Figure S4.

Supplementary Figure S5.

Supplementary Legends.

Supplementary Table S1.

Supplementary Table S2.

Supplementary Table S3.

Supplementary Table S4.

Supplementary Table S5.

Supplementary Table S6.

Supplementary Table S7.

Supplementary Table S8.

Supplementary Table S9.

Supplementary Table S10.

Supplementary Table S11.

Supplementary Table S12.

## Data Availability

These sequence data for this study can be found in the European Nucleotide Archive (ENA) database under accession number PRJEB31936: https://www.ebi.ac.uk/ena/browser/view/PRJEB31936.
