# Supplementary figures and images for: Paraburkholderia phytofirmans PsJN colonization of rice endosphere triggers an atypical transcriptomic response compared to rice native Burkholderia s.l. endophytes
Source: Sci Rep. 2023 Jul 3;13:10696. doi: 10.1038/s41598-023-37314-7 (PMC10317989; doi:10.1038/s41598-023-37314-7)

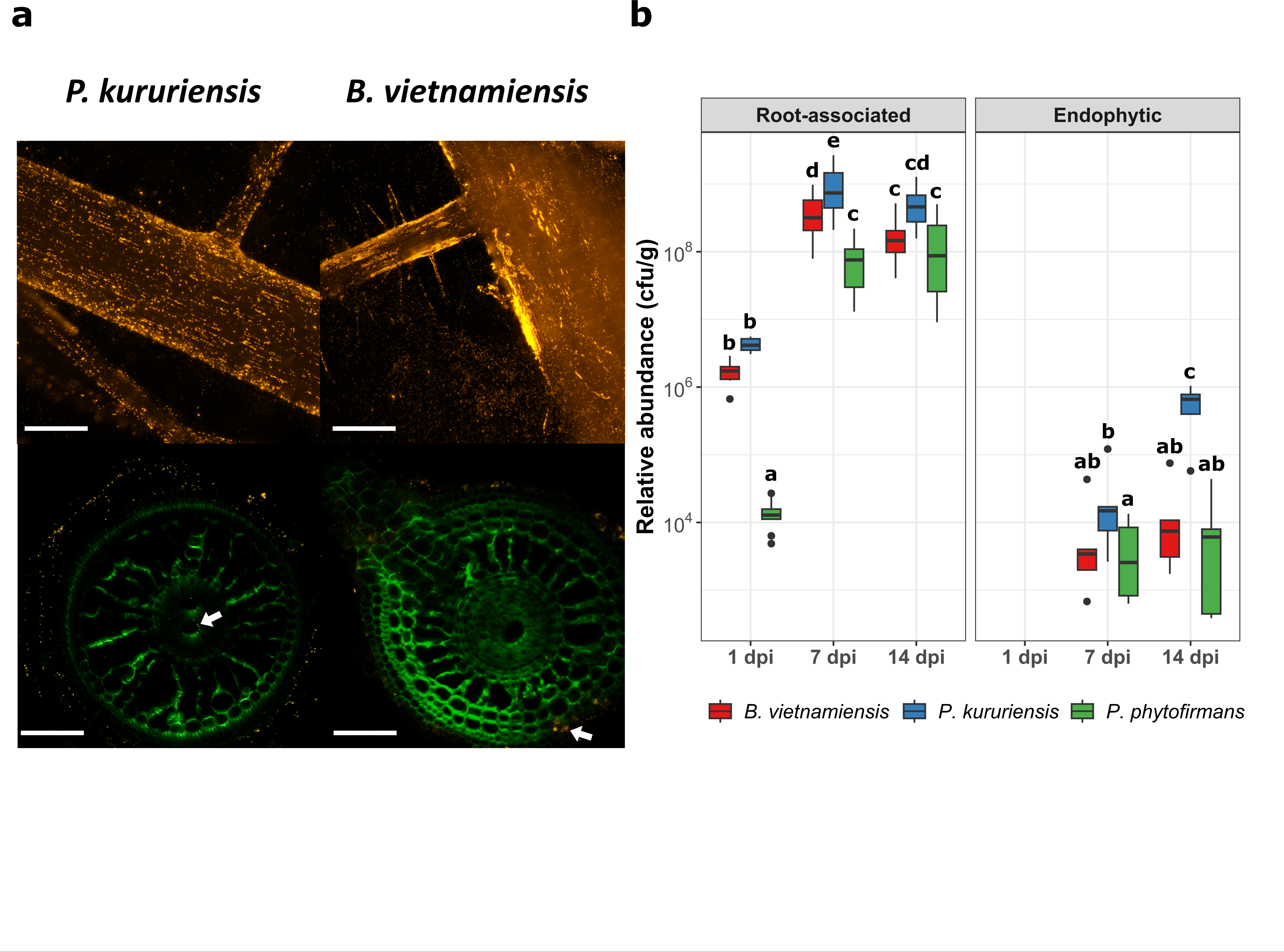

Supplement: Supplementary file 1 — Supplementary Figure S1. [file 41598_2023_37314_MOESM1_ESM.png]

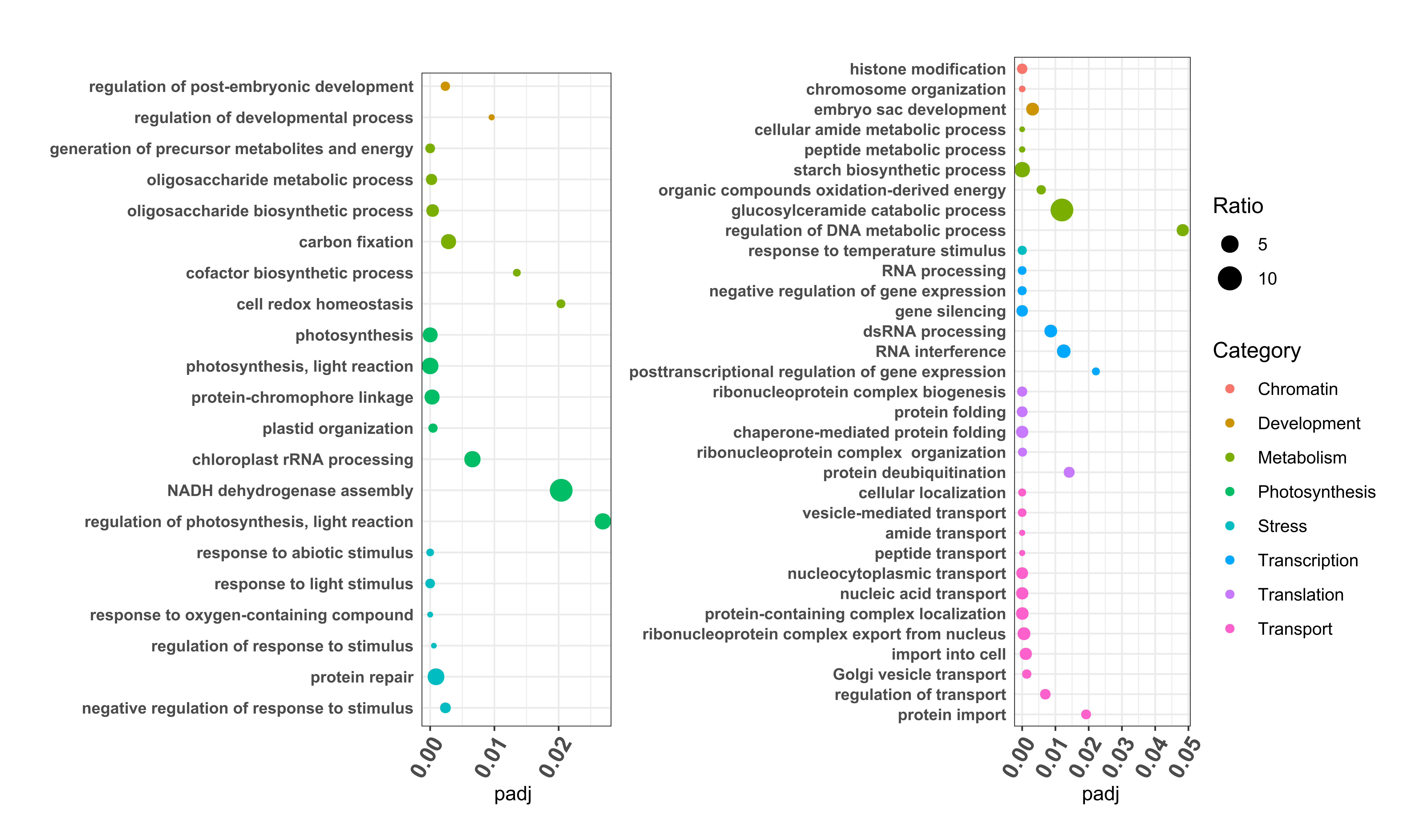

Supplement: Supplementary file 2 — Supplementary Figure S2. [file 41598_2023_37314_MOESM2_ESM.png]

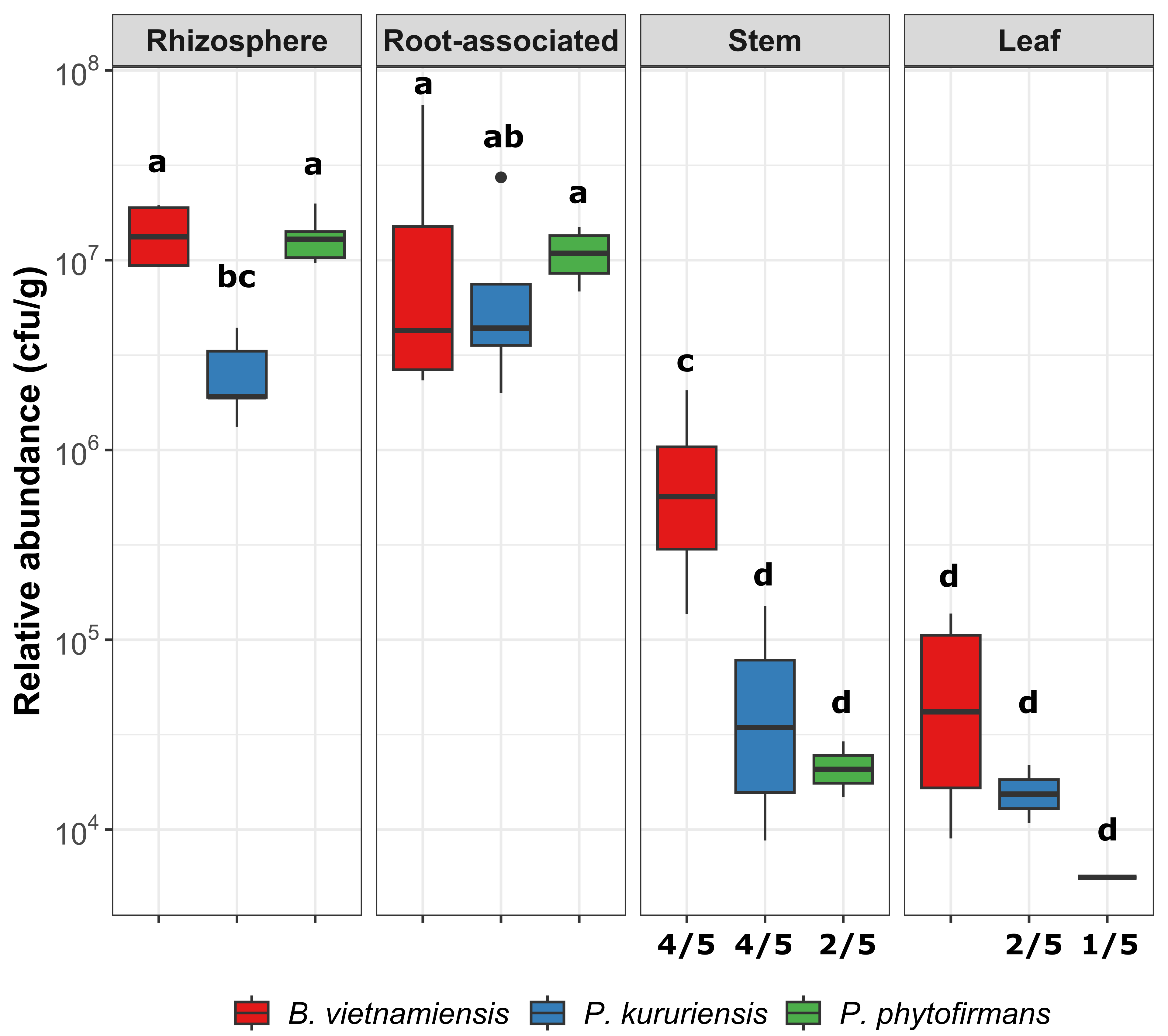

Supplement: Supplementary file 3 — Supplementary Figure S3. [file 41598_2023_37314_MOESM3_ESM.tiff]

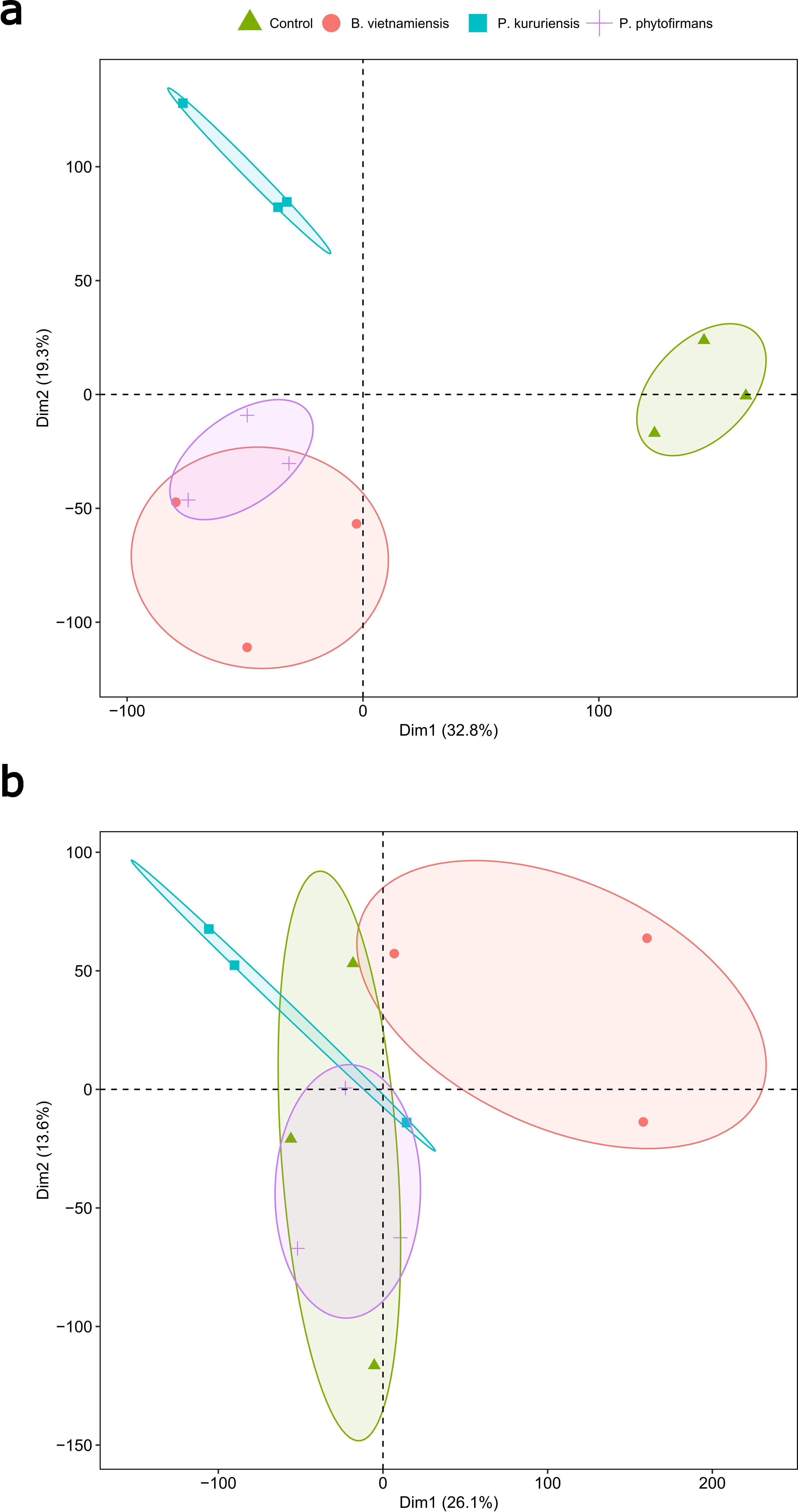

Supplement: Supplementary file 4 — Supplementary Figure S4. [file 41598_2023_37314_MOESM4_ESM.jpg]

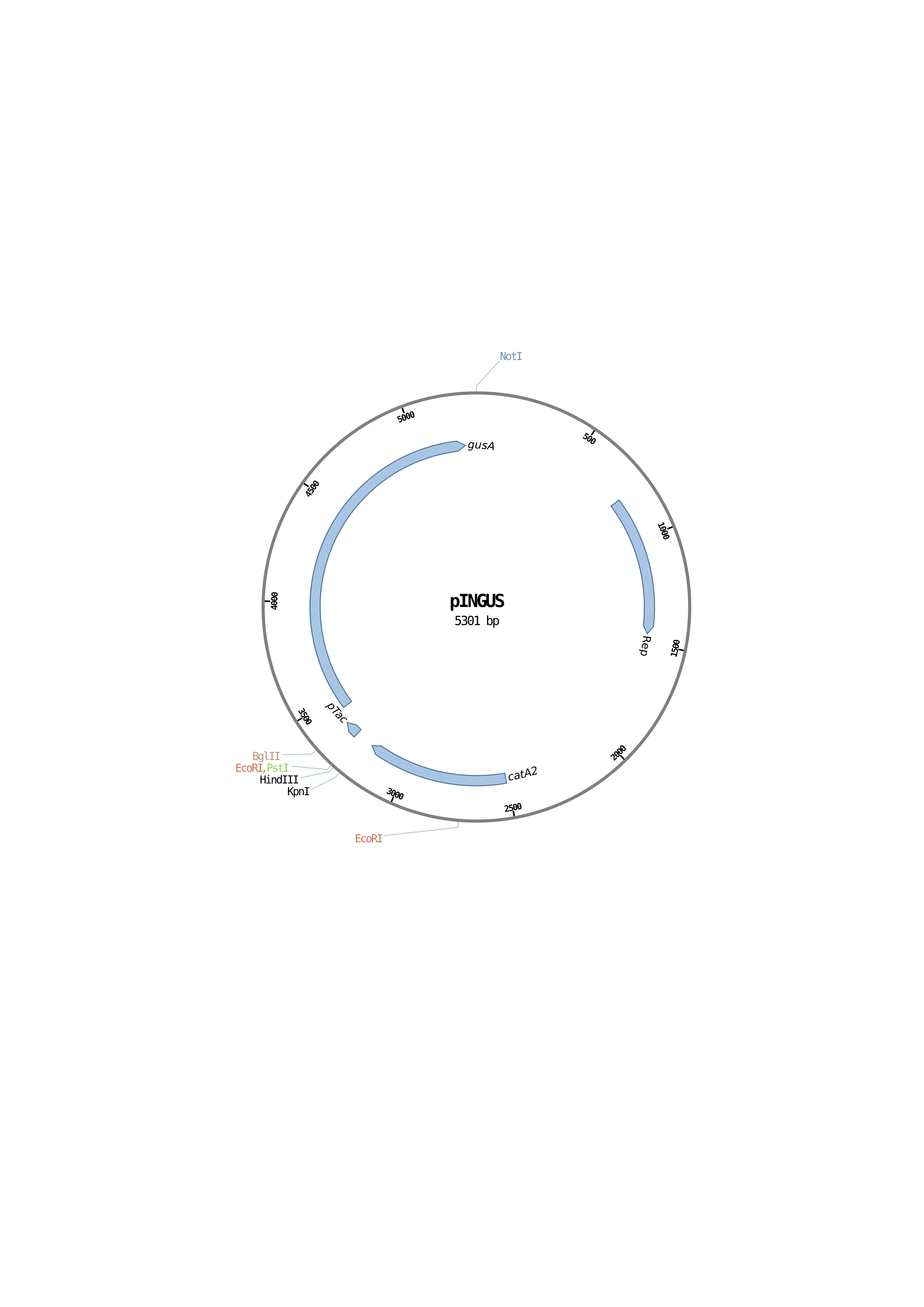

Supplement: Supplementary file 5 — Supplementary Figure S5. [file 41598_2023_37314_MOESM5_ESM.png]
