# Supplementary material for: Paraburkholderia phytofirmans PsJN colonization of rice endosphere triggers an atypical transcriptomic response compared to rice native Burkholderia s.l. endophytes
Source: Sci Rep. 2023 Jul 3;13:10696. doi: 10.1038/s41598-023-37314-7 (PMC10317989; doi:10.1038/s41598-023-37314-7)
Supplement: Supplementary file 6 — Supplementary Legends. [file 41598_2023_37314_MOESM6_ESM.docx]

**Supplementary Figure 1.** Endophytic colonization of rice roots by PsJN. (a) Epifluorescence and confocal microscopy pictures of the of the surface and crosssection of rice primary root respectively at 7 and 17 days post-inoculation by Pk::pIN29 and Bv::pIN29 cells. White bars represent 100 μm. (b) Population dynamics of DsRed-tagged PsJN associated with rice roots. The data reported are the median of bacterial population size from 9 plants for 1 dpi and 18 plants -conducted in two independent experiments- for 7 and 14 dpi. For the endophytic compartment the data correspond to 5 biological replicates. Letters indicate significantly different groups (P < 0.05) according to Tukey’s post-hoc test performed separately on for each compartment on a negative binomial zero-inflated model.

**Supplementary Figure 2.** Enrichment analysis of Biological Process GO terms of PsJN-treated rice leaf transcriptome. Y-axis corresponds to the significantly enriched GO terms (FDR < 0.05 and an enrichment ratio threshold of 2), X-axis corresponds to the adjusted p-value, the enrichment ratio represent by the size of the dots corresponds to the ratio between the proportion of genes related to the given GO term in the transcriptome and the proportion of genes related to the given GO term in the rice genome. Left and right panels respectively correspond to up- and down-regulated DEGs.

**Supplementary Figure 3.** Colonization of soil-grown rice plants by PsJN, Pk and Bv. Relative abundance of DsRed-tagged PsJN, Pk and Bv isolated from rice rhizosphere, roots, stem and leaves. The data reported are the median of bacterial population size from 5 plants at 15 dpi with 10^8^ bacterial cells one week after sowing. Letters indicate significantly different groups (P < 0.05) according to Tukey’s post-hoc test performed on negative binomial zero-inflated model.

**Supplementary Figure 4.** Principal component analysis of the transcriptome of rice in response to PsJN, Pk and Bv. Principal component analysis of the normalized number of reads mapped per gene in leaves (a) and in roots (b) in response to PsJN, Pk and Bv and mock (control).

**Supplementary Figure 5.** Physical maps of pINGUS plasmid constructed in this study. Abbreviations: catA2, chloramphenicol resistance gene; Rep, required for replication; gusA, gene encoding a β-glucuronidase; pTac, tac promoter sequence.
